# On-chip micro-ring resonator array spectrum detection system based on convex optimization algorithm

**DOI:** 10.1515/nanoph-2022-0672

**Published:** 2023-01-20

**Authors:** Xinyang Chen, Xuetao Gan, Yong Zhu, Jie Zhang

**Affiliations:** The Key Laboratory of Optoelectronic Technology & System, Education Ministry of China, Chongqing University, 400044, Chongqing, People’s Republic of China; Key Laboratory of Light-Field Manipulation and Information Acquisition, Ministry of Industry and Information Technology, and Shaanxi Key Laboratory of Optical Information Technology, School of Science, Northwestern Polytechnical University, Xi’an 710072, China

**Keywords:** convex optimization algorithm, MRRAS, spectrum reconstruction, waveguide transmission theory

## Abstract

We propose an all on-chip micro-ring resonator array spectrum detection system (MRRAS). Micro-ring resonator array as the core is used to construct the transmission matrix of the system. The theoretical analysis of the spectrum detection system is completed with waveguide transmission theory and spectrum construction method based on convex optimization algorithm. In the experiment, we obtain the priori information of the transmission matrix of the system, then detect the output intensity of unknown spectrum through MRRAS, and construct the under-determined matrix equations when the number of micro-rings is much smaller than that of reconstructed wavelengths. Convex optimization algorithm is employed to obtain the least norm solution of the under-determined matrix equations, which enables fast spectrum reconstruction. The experimental results show that the spectrum detection system is constructed using three micro-ring resonators with 4 μm radius, enabling the compact footprint. In addition, the silicon nitride based photonic platform is fully compatible with standard complementary metal oxide semiconductor (CMOS) processes. The system operating bandwidth is more than 12 nm and the resolution is better than 0.17 nm.

## 1 Introduction

The miniaturization of optical spectrometers, especially in chip-integrated architecture, is an active field of research, which could facilitate the development of chemical and biological analysis, environmental monitoring, and hyperspectral imaging [1–6]. Especially for airborne and spaceborne astrophotonic sensing applications, it is important to reduce the size, weight, and complexity of the spectrometer through compact integration [7–9]. Over the past few years, silicon nitride has been one of the main platforms for building integrated photonic circuits [10]. It has a higher refractive index than silicon oxide and a wide band gap (e.g.∼5.1 eV), with transparent windows extending from infrared to visible and even ultraviolet. These advantages promise the implementation of long optical transmission delay lines and compact device footprints in silicon nitride platform, which is essential for achieving high spectral resolution in various types of integrated spectrometers.

Numerous approaches have been taken to achieve high performance on-chip integrated spectrometers. Currently, on-chip spectrometers are mainly divided into split-channel dispersive spectrometers and digital spectrometers based on Fourier transform. Dispersion elements, such as array waveguide grating (AWG) [11–13] and planar concave grating [14], are widely used in traditional dispersive spectrometers. Although these spectrometers can theoretically provide high optical resolution for broadband incident spectral signals, these schemes are achieved at the expense of introducing a large number of channels and detectors, resulting in considerably large footprint, high insertion loss, and greatly reduced signal-to-noise ratio (SNR). Recently, researchers proposed to use high Q-value micro-ring resonator (MRR) filter arrays for spectrum detection through split channels [15]. The precondition is that MRR filter arrays have very low loss, so a high Q-value can be obtained with a smaller coupling factor [16]. However, due to the unavoided fabrication imperfections, material absorption and bending scattering, it is still difficult to achieve ultralow waveguide losses and the actual output position of each channel is biased [17].

In contrast, due to Fellgett’s advantage in terms of high SNR, Fourier transform spectrometers (FTS) have been extensively investigated, such as spatial heterodyne spectrometers (SHS) [18–21] and stationary-wave integrated Fourier transform spectrometers (SWIFT) [22–24]. It has been reported that SHS use Mach–Zehnder Interferometer (MZI) arrays to uniformly sample each point in the interferogram [18], achieving a resolution of ∼0.04 nm, but it requires a large number of MZI arrays. SWIFT uses mirror reflection to form standing waves with a resolution of 4 nm at the central wavelength of 1500 nm [23]. However, in order to satisfy the Nyquist–Shannon criterion, the distance between the two detectors must be less than *λ*/4*n*_eff_ (the wavelength *λ*, the effective refractive index *n*_eff_). This requirement for a typical micro-sized charge coupled device (CCD) is impossible. To reduce the number of detection system arrays.

David Pohl et al. [25] and Miguel Montesinos-Ballester et al. [20] continuously tune the optical path delay (OPD) between the two pathways of the incident spectral signals in the spectrometer by electrical and thermal method respectively, and detect the interference intensity at each OPD to obtain the interferograms of the incident spectral signals. Then, the incident spectral signals with a high-resolution and broadband are reconstructed using Fourier transform technology. Similarly, Zheng et al. [26] obtained the interferograms by continuously thermal tuning one arm of MZI. Limited by maximum heating power, high resolution (long waveguide delay line) Fourier transform spectrometer is difficult to achieve. Therefore, it is still challenging to achieve a high performance, small footprint and low power consumption integrated spectrometer.

In this paper, we utilize the convex optimization algorithm, which is widely used in machine learning, to optimize the least-norm problems established by unknown spectrum through MRR array spectrum detection system (MRRAS), so as to achieve spectrum reconstruction. Note that on-chip integration of Raman sensing with trace substance detection capability and spectrum detection systems is a hot issue. We would like to utilize the 785 nm laser as the excitation of Raman signal to construct an on-chip Raman sensor. Therefore, the MRRAS operating bandwidth should be combined with the Stoke Raman spectrum (excitation wavelength is 785 nm). The operating wavelength range of the spectrum detection system is ∼800 nm. Based on the experimental results, we realize the reconstruction of typical spectrum in the spectral range exceeding 12 nm.

## 2 Theoretical analysis of MRRAS

### 2.1 Spectrum detection system

**Figure 1:**
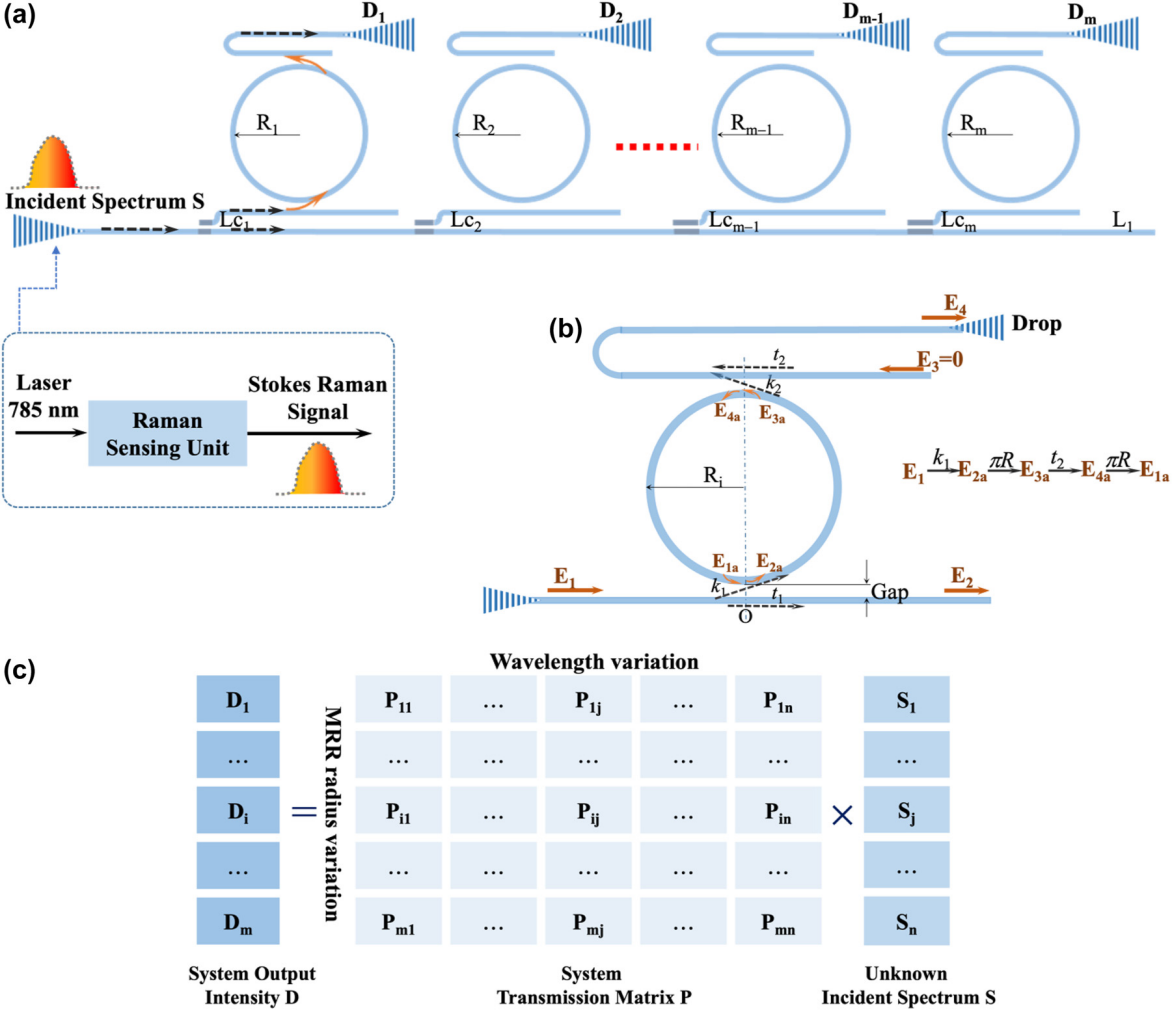
Schematic diagram of (a) micro-ring resonator array spectrum detection system and *L*_*c*_ are direct waveguide couplers. (b) Add-drop micro-ring resonator. (c) System matrix equation constructed by micro-ring resonator array. Note that the incident spectrum *S* comes a Raman sensing unit by an excitation light of 785 nm laser, so the operating wavelength range of the spectrum detection system is ∼800 nm.

The schematic of MRRAS is shown in Figure 1(a). The core of the system is an MRR array composed of *m* MRRs. The MRRs have slightly varied radius of *R*_1_, *R*_2_, …, *R*_*m*_. The incident light with unknown spectrum *S* is coupled into the waveguide *L*_1_ by a grating coupler and passes into the MRR array channels with the same intensity ratio. Then, we detect the intensity information of the unknown spectrum at each MRR output and record it as *D*_1_, *D*_2_, …, *D*_*m*_.

As shown in Figure 1(b), we choose add-drop MRR and the system matrix equation constructed is shown in Figure 1(c). The detected intensity information *D* of an incident spectrum *S* (*λ*) passing through MRR arrays with transmission matrix *P* (*λ*) can be mathematically written as:

(1) $$D_{i}=\int P_{i}\left(\lambda \right)\times S\left(\lambda \right)d\lambda$$

where *i* = 1, 2, …, *m*, *λ* is the wavelength. *P*_*i*_ (*λ*) represents the drop signals of the *i*th MRR, expressed as [16]:

(2) $$P_{i}=\frac{\left(1-t_{1}^{2}\right)\left(1-t_{2}^{2}\right)\alpha_{\text{mrr}}}{1-2t_{1}t_{2}\alpha_{\text{mrr}}\cos\left(k_{0}n_{\text{eff}}2\pi R_{i}\right)+{\left(t_{1}t_{2}\alpha_{\text{mrr}}\right)}^{2}}$$

where *k*_0_ = 2*π*/*λ*, *α*_mrr_ is the loss factor of MRR and *n*_eff_ is the effective refractive index of the waveguide material. *t*_1_ and *t*_2_ are the MRR transmission coefficient and *t*_1_ = *t*_2_. In Eq. (1), *λ* represents the ideal continuous wavelength variable; however, when processing an actual spectral signal, *λ* is discrete data. Therefore, the detected intensity *D*_*i*_ should be expressed as:

(3) $$D_{i}=\overset{n}{\underset{j=1}{\sum }}P_{i}\left(\lambda_{j}\right)\times S\left(\lambda_{j}\right)$$

where *j* = 1, 2, …, *n* and *n* is the number of wavelength. Therefore, when the system contains *m* MRRs and *n* discrete wavelength points, the relationship of the matrix dimensions is constructed as shown in Eq. (4).

(4) $$D_{m\times 1}=P_{m\times n}\times S_{n\times 1}$$

When the system contains *n* discrete wavelength points and the bandwidth of the incident spectrum is Bw, the wavelength interval can be express as Δ*λ* = Bw/*n*. Generally, the smaller the wavelength interval, the higher the system resolution can be obtained.

### 2.2 Reconstruction algorithm

According to Eq. (4), when *m* = *n*, matrix *P* is square matrix and can be solved directly. Xia et al. [27]. used 84 MRRs to construct a square matrix (transmission matrix) to reconstruct an unknown spectrum with a resolution of 0.6 nm and an operating bandwidth of 50 nm, but the footprint of the spectrometer is ∼1 mm^2^. In order to reduce the system footprint and the insertion loss induced by the system complexity, we expect to use fewer MRRs to reconstruct spectrum, that is, *m*<<*n*. Then, solving Eq. (4) is a common problem to solve under-determined matrix equations.

Equation (4) has an infinite number of solutions, so additional constraints are required. Constraints can be introduced to construct a least-squares optimization algorithm to solve the problem, or a linear programming algorithm can be used to solve the problem, where both least-squares and linear programming are special convex optimization problems. In this paper, we establish a classical convex optimization algorithm based on the least ℓ _2_-norm to solve the under-determined matrix equations [28]:

(5) $$\begin{array}{c} \min{\left\|x\right\|}_{2}\\ s.t.\;D=P\times x\;x\geq 0 \end{array}$$

where *x* ∈ **R** ^*n*^, *P* ∈ **R** ^*m*×*n*^, *D* ∈ **R** ^*m*^. ℓ _2_-norm of vector *x* is defined as:

(6) $${\left\|x\right\|}_{2}=\sqrt{\overset{n}{\underset{j=1}{\sum }}x_{j}^{2}}$$

In the field of norm approximation [28], comparing the ℓ _1_-norm with the ℓ _2_-norm, we know that:

(1) The ℓ _1_-norm penalty puts the most weight on small residuals and the least weight on large residuals.
(2) The ℓ _2_-norm penalty puts very small weight on small residuals, but strong weight on large residuals.

This means that in ℓ _1_-norm approximation, we typically find that many of the equations are satisfied exactly. The similar effect occurs in the least-norm problems. The least ℓ _1_-norm tends to produce sparse solutions, and the least ℓ _2_-norm tends to give continuous and balanced solutions. At the same time, we use the regularization algorithm in machine learning to optimize the solutions of under-determined matrix equations. The simplest way is to introduce the smoothing function [28]:

(7) $$B=\left[\begin{array}{ccccccc} -1 & 1 & 0 & \ldots & 0 & 0 & 0\\ 0 & -1 & 1 & \ldots & 0 & 0 & 0\\ \vdots & \vdots & \vdots & \vdots & \vdots & \vdots & \vdots \\ 0 & 0 & 0 & \ldots & -1 & 1 & 0\\ 0 & 0 & 0 & \ldots & 0 & -1 & 1 \end{array}\right]$$

where *B* ∈ **R** ^(*n*−1)×(*n*)^ is the bidiagonal matrix. Based on the above analysis, the convex optimization algorithm based on least norm is constructed:

(8) $$\begin{array}{c} \min{\left\|x\right\|}_{2}+\gamma_{1}{\left\|x\right\|}_{1}+\gamma_{2}{\left\|B\times x\right\|}_{2}\\ s.t.\;D=P\times x\;x\geq 0 \end{array}$$

where ‖*B* × *x*‖ is smoothing function. ℓ _1_-norm of vector *x* is defined as:

(9) $${\left\|x\right\|}_{1}=\overset{n}{\underset{j=1}{\sum }}\left|x_{j}\right|$$

Through the effective regulation coefficients *γ*_1_ and *γ*_2_, ℓ _1_-norm, ℓ _2_-norm, and smoothing function together to optimize the solutions of the under-determined matrix equations, that is, the reconstruction results.

## 3 Design of the MRRAS and simulations

As shown in Figure 1(c), transmission matrix *P* contains *m* row vectors, which ideally should be linear independent (row full rank). During the design process, the micro-ring resonance wavelengths are set at equal intervals, and there is only one resonance peak for each MRR within the operating bandwidth. The drop spectrum intensity overlay of each MRR is considered as the wavelength weights within the operating bandwidth. Ideally, the smooth weight value is beneficial to obtain better reconstruction results. The number of MRR depends on the full width at half maximum (FWHM) of the MRR and the unknown spectrum operating bandwidth. Smoother weight values can be obtained by increasing the number of MRRs, and more MRRs will increase optimization constraints and obtain more accurate reconstructed spectrum, but a large number of MRRs can cause spectrum detection system complexity. So we consider that smooth weights should be achieved with a small number of MRR, and then optimization algorithms can be used to improve system performance.

**Figure 2:**
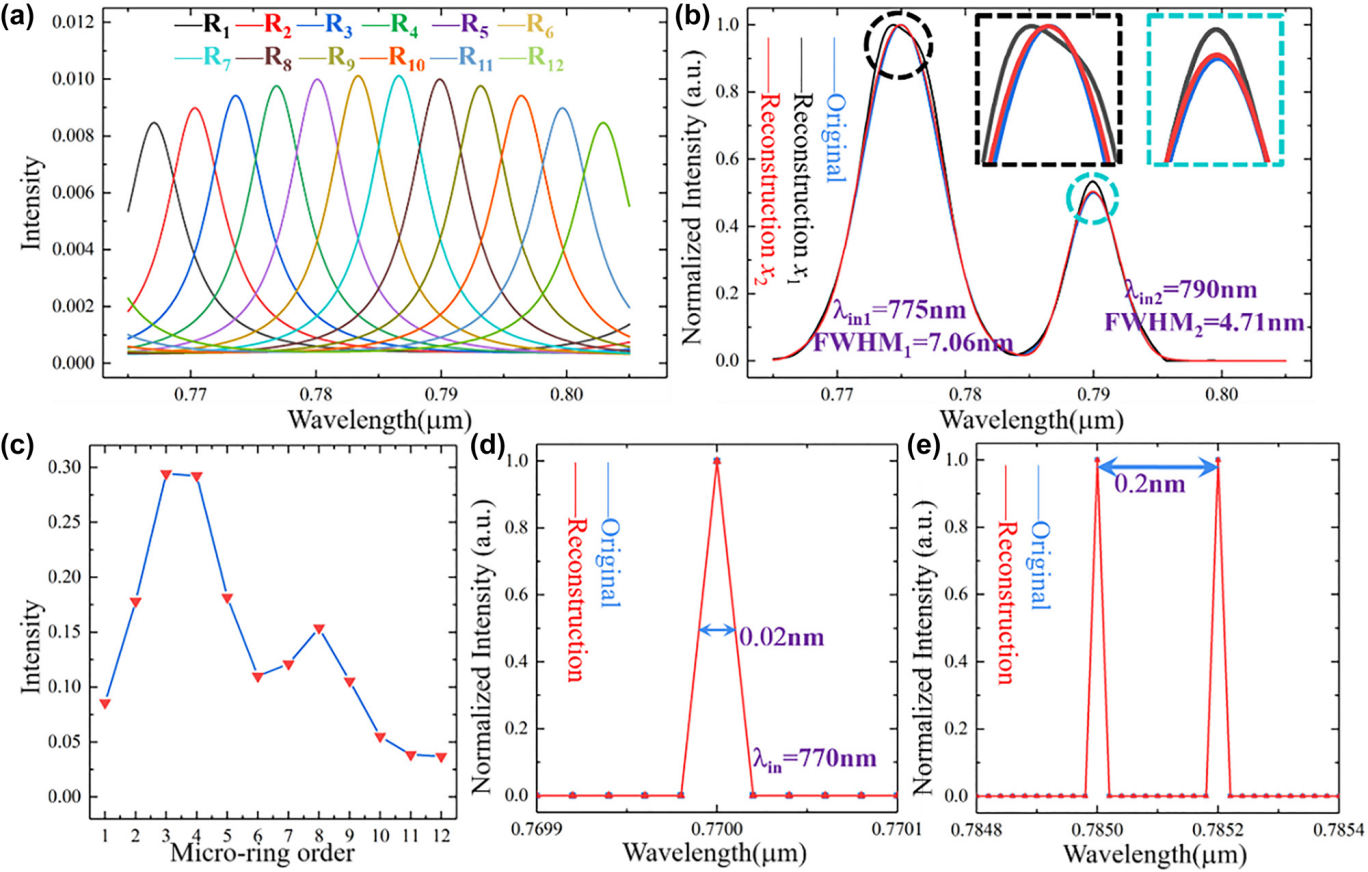
Unknown spectrum reconstruction process. (a) MRRs Drop spectrum under modulation. (b) Reconstruction results of double broadband spectrum. (c) Output values of incident spectrum at each micro-ring. (d) and (e) Reconstruction results of the single narrowband spectrum and two adjacent narrowband spectrums.

We use the CVX toolbox to establish convex optimization algorithm and simulate the unknown spectrum reconstruction process in Matlab environment. The number of MRRs is 12, the effective refractive index of silicon nitride waveguide is *n*_eff_ = 2.23, the MRR coupling coefficient is *κ* ^2^ = 0.3, the transmission coefficient is *t* ^2^ = 0.7, the loss factor is *α*_mrr_ = 0.95, the spectrum ranges from 765 nm to 805 nm and the wavelength interval Δ*λ* is 0.02 nm. We adjust the MRR radius to make the drop spectrum peak wavelengths of each MRR evenly distributed in the operating bandwidth. The MRR radius is *R*_1_ = 985.33 nm, *R*_2_ = 989.54 nm, *R*_3_ = 993.74 nm, *R*_4_ = 997.95 nm, *R*_5_ = 1002.15 nm, *R*_6_ = 1006.35 nm, *R*_7_ = 1010.56 nm, *R*_8_ = 1014.76 nm, *R*_9_ = 1018.97 nm, *R*_10_ = 1023.17 nm, *R*_11_ = 1027.38 nm and *R*_12_ = 1031.58 nm, respectively. MRR Drop spectra modulated by a grating coupler are shown in Figure 2(a), where the center wavelength of the grating coupler modulated signal center wavelength is 785 nm and FWHM is 24.8 nm. Here, the incident spectrum consists of two broad Gaussian spectrum. The incident center wavelength is *λ*_in1_ = 775 nm with FWHM_1_ = 7.06 nm and *λ*_in2_ = 790 nm with FWHM_2_ = 4.71 nm.

The system output values corresponding to the incident spectrum are shown in Figure 2(c) and the reconstructed results are shown in Figure 2(b). The reconstructed spectrum *x*_1_ (black line) is calculated based on Eq. (5). The reconstruction error *ϖ* is 1.27% and the maximum error *ϖ*_max_ is 7.85%. (*ϖ* represents the average result of absolute errors for all reconstruction points). The illustrations show the reconstructed results at the center wavelengths of the incident spectrum. As a comparison, the reconstructed spectrum *x*_2_ (red line) obtained by the convex optimization algorithm with Eq. (8) is almost identical to the incident spectrum (*ϖ* = 0.58% and *ϖ*_max_ = 2.72%).

The comparison of the reconstruction errors clearly reflects that the reconstruction spectra can be optimized by using ℓ _1_-norm, ℓ _2_-norm, and smoothing function, which correspond to the results of the theoretical analysis section. This means that it is possible to obtain the desired reconstructed spectrum when the number of MRRs is insufficient. System resolution experiments are performed and the results are shown in Figure 2(d) and (e). We successfully reconstructed narrowband spectral signals with different center wavelengths and FWHM less than 0.02 nm. In this case, the introduced parameter *F* represents the system resolution, and the resolution is better than 0.02 nm. The reconstruction spectrum of the two adjacent narrowband spectra (*F* = 0.02 nm and center wavelength spacing is 0.2 nm) are shown in Figure 2(e). The simulation results verify that MRRAS can be used to detect on-chip sensor system composed of high Q-value devices (MRR and Fabry–Perot cavity).

## 4 Experiment and results

### 4.1 MRR characterization

**Figure 3:**
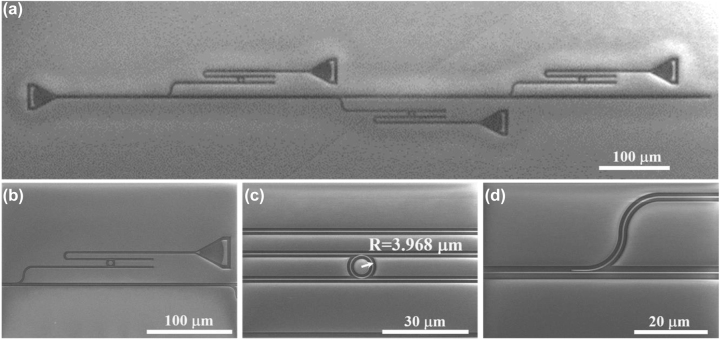
The SEM images of (a) the micro-ring resonator array. (b) A single unit in MRRAS. (c) The micro-ring resonator with *R* = 3.968 μm. (d) A directional coupler.

According to the above analysis and verification, we fabricated the micro-ring array spectrum detection system using e-beam lithography (EBL) and inductively coupled plasma (ICP) etching. The scanning electron microscope (SEM) image of MRRAS is shown in Figure 3(a). To reduce the difficulty of the fabrication procedure and improve the spectrum quality, we compared the Drop spectra with different micro-ring radii and finally chose 4 μm micro-ring radius with 12 nm operating bandwidth. The spectrum detection system consists of three integrated units as shown in Figure 3(b). Employ three MRRs to construct the transmission matrix, where the micro-ring radii are *R*_1_ = 3.968 μm, *R*_2_ = 3.990 μm, and *R*_3_ = 4.011 μm. Figure 3(c) shows a micro-ring resonator with a radius of 3.968 μm and a coupling gap of about 70 nm. Each MRR unit is integrated by a directional coupler and the coupling lengths are *Lc*_1_ = 3.8 μm, *Lc*_2_ = 4.8 μm, and *Lc*_3_ = 9.6 μm, respectively. The directional coupler with *Lc*_1_ = 3.8 μm is shown in Figure 3(d), and the coupling gap is 150 nm to ensure small efficiency fluctuation. In this work, we construct the system incident spectrum using the super-continuum sources combined with an Acousto-optic tunable filter (AOTF). Since the FWHM of AOTF output spectrum is about 2 nm–4 nm, the calibration error of each sampling wavelengths of the system transmission matrix is large by using this method. Therefore, we use the spectrometer to obtain the prior information of the system transmission matrix directly.

### 4.2 Broadband spectrum reconstruction

**Figure 4:**
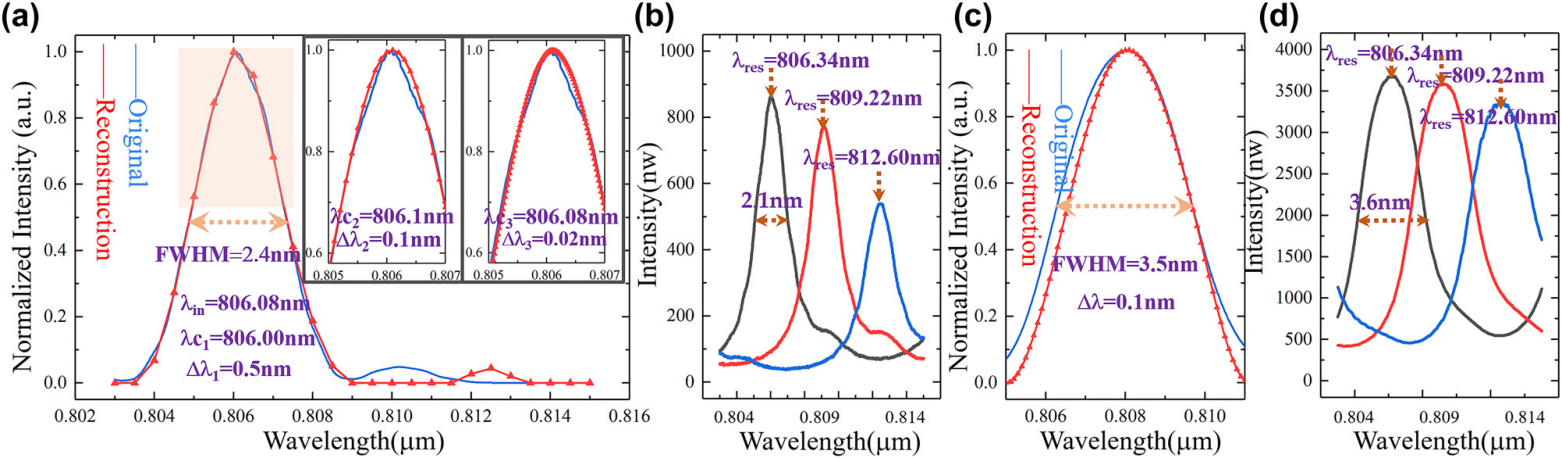
The reconstructed results of broadband spectrum with three micro-rings. (a) The reconstructed spectrum at different wavelength intervals with *λ*_in_ = 806.08 nm and FWHM = 2.4 nm. (b) The system transmission spectra with FWHM of 2.1 nm. (c) The reconstructed spectrum with *λ*_in_ = 808.06 nm and FWHM = 3.5 nm. (d) The system transmission spectra with FWHM of 3.6 nm.

Set the operating bandwidth range from 803 nm to 815 nm, the reconstructed results of the incident spectrum with *λ*_in_ of 806.08 nm and FWHM of 2.4 nm are shown in Figure 4(a). The transmission spectra are shown in Figure 4(b). The reconstruction error is small, where *ϖ* is 1.68% and *λ*_*c*1_ = 806 nm when the matrix *P* wavelength sampling interval Δ*λ* is 0.5 nm. The illustration also shows the corresponding reconstruction results when the matrix *P* wavelength sampling intervals Δ*λ* are different. It can be seen that when Δ*λ* is smaller (Δ*λ* = 0.1 nm and 0.02 nm), more details can be observed (*λ*_*c*2_ = 806.1 nm and *λ*_*c*3_ = 806.08 nm). In addition, the optimized coefficients are *γ*_1_ = 10 and *γ*_2_ = 26 (Δ*λ* = 0.5 nm), *γ*_1_ = 10 and *γ*_2_ = 260 (Δ*λ* = 0.1 nm), and *γ*_1_ = 10 and *γ*_2_ = 2600 (Δ*λ* = 0.02 nm), respectively. The optimized coefficients *γ*_1_ and *γ*_2_ depend on the MRR used to form the transmission matrix *P* and wavelength interval Δ*λ*, and it can be pre-determined by sending some known spectrum to the system. At the same time, the reconstructed results of the incident spectrum with different FWHM and central wavelength are shown in Figure 4(c), where the transmission spectra are shown in Figure 4(d). The radii of the MRRs are the same in Figure 4(b) and (d). Different resolution parameters are selected in the spectrometer (AQ6370D) to obtain transmission spectra with different FWHM. Then, due to different transmission matrix *P*, the optimized coefficients need adjustment, where *γ*_1_ = 10 and *γ*_2_ = 75 (Δ*λ* = 0.1 nm). The incident center wavelength is 808.06 nm and the reconstructed center wavelength is 808.1 nm, where Δ*λ* is 0.1 nm and *ϖ* is 4.6%. The weights of MRR arrays are more balanced in the central of the operating bandwidth and more accurate reconstructed results are observed, that is, under the optimized conditions of the least norm, the reconstructed spectrum tends to be heavily weighted.

### 4.3 Double broadband spectrum reconstruction

**Figure 5:**
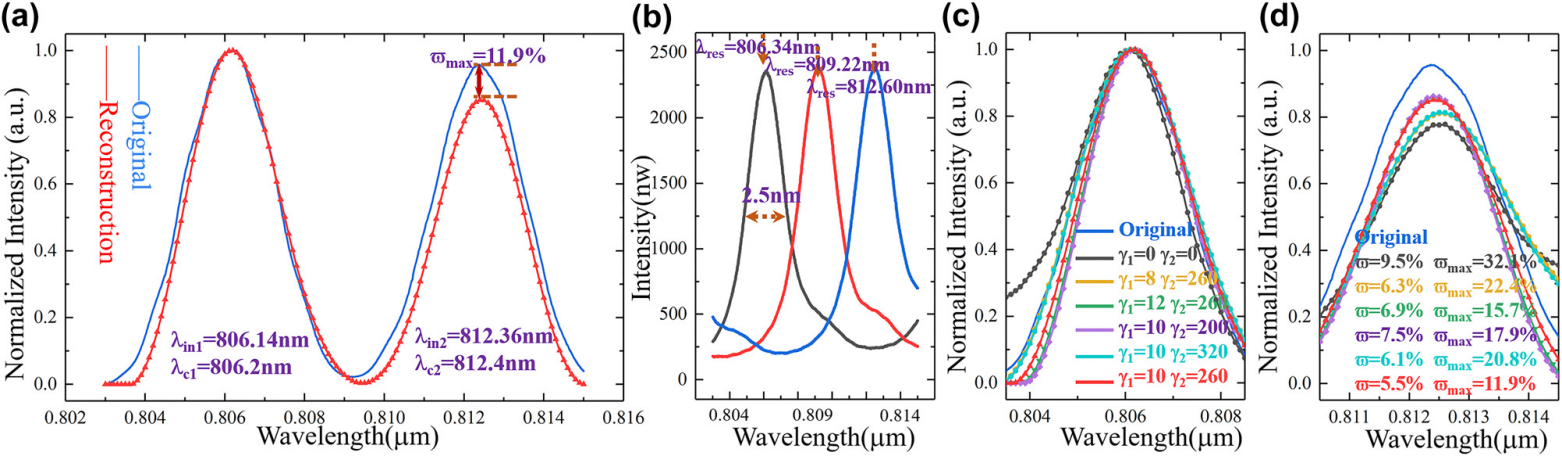
The reconstructed results of double broadband spectrum with three micro-rings. (a) The optimized reconstructed results of double broadband spectrum. (b) The system transmission spectra with FWHM of 2.5 nm. (c) and (d) The reconstructed spectrum with different optimized coefficients (*γ*_1_ and *γ*_2_) and the corresponding reconstructed spectrum error is shown (*ϖ* and *ϖ*_max_). The spectrum with blue line is the original incident spectrum and the red line is the optimized reconstructed spectrum.

Further, we reconstruct the double broadband spectrum as shown in Figure 5(a), where the incident spectrum center wavelengths are *λ*_in1_ = 806.14 nm and *λ*_in2_ = 812.36 nm, and the reconstructed spectrum center wavelengths are *λ*_*c*1_ = 806.2 nm and *λ*_*c*2_ = 812.4 nm. The reconstruction error is 5.5% and the maximum error is 11.9%, where optimization coefficients are *γ*_1_ = 10 and *γ*_2_ = 260. The transmission spectra are shown in Figure 5(b). In addition, the importance of selecting the correct optimization coefficients for Eq. (8) is demonstrated experimentally. The reconstructed results near the two main peaks of the original spectrum (as shown in Figure 5(a)) under different optimized coefficients (*γ*_1_ and *γ*_2_) are shown in Figure 5(c) and (d), respectively. The original spectrum (blue line), no optimized reconstructed spectrum (*γ*_1_ = 0, *γ*_2_ = 0), under-optimized reconstructed spectrum (*γ*_1_ = 8, *γ*_2_ = 260 and *γ*_1_ = 10, *γ*_2_ = 200), over-optimized reconstructed spectrum (*γ*_1_ = 12, *γ*_2_ = 260 and *γ*_1_ = 10, *γ*_2_ = 320), and optimized reconstructed spectrum (*γ*_1_ = 10, *γ*_2_ = 260) are displayed and reconstructed spectrum error (*ϖ* and *ϖ*_max_) are compared, where the operating bandwidth range from 803 nm to 815 nm. Here, the optimized reconstructed spectrum (red line) shows the minimum reconstructed error (*ϖ* = 5.5% and *ϖ*_max_ = 11.9%) and the minimum reconstructed center wavelength drift (almost exactly the same). However, there is still some reconstructed intensity error at the center wavelength *λ*_in2_, which is due to the drift of the wavelength and intensity of the incident spectrum during the experiment (maximum intensity drift is close to 10%) as well as the spatial position errors of the grating couplers. It is difficult to control that the spatial position of the grating coupler is exactly the same during the acquisition of transmission matrix and spectrum reconstruction. In the reconstruction process, when the grating coupler efficiency at a certain wavelength becomes higher than that at the acquisition of the priori matrix *P*, the reconstructed spectrum intensity will be decreases.

**Figure 6:**
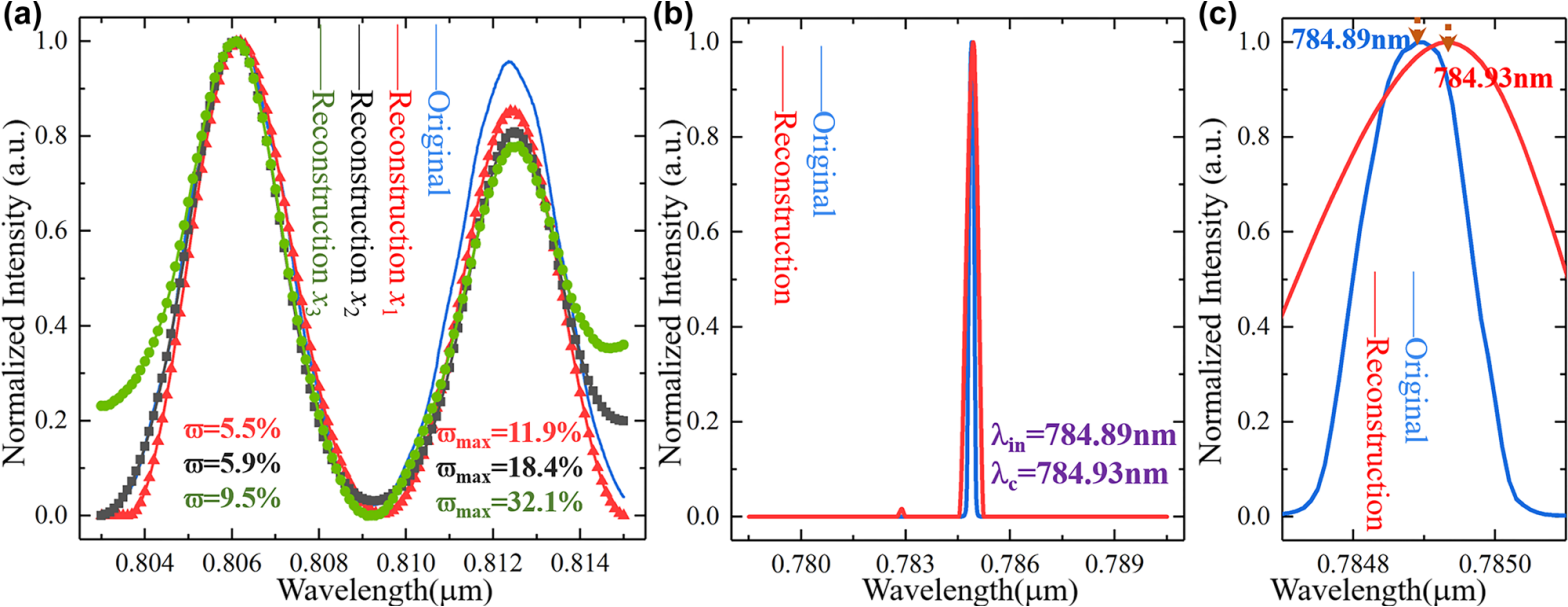
The reconstructed spectra by different convex optimization algorithm and single wavelength characterization. (a) The reconstructed spectra *x*_1_,* x*_2_, *x*_3_ by the least norm, norm approximation and least-squares optimization algorithm. (b) The reconstructed spectrum of laser signal with the center wavelength of 784.89 nm and FWHM of 0.17 nm. (c) Reconstructed results at the center wavelength, where the reconstructed wavelength error is 0.04 nm.

Here, we compare the reconstructed spectra (*x*_1_, *x*_2,_ and *x*_3_) by different convex optimization algorithm with the same experimental data. As shown in Figure 6(a), the reconstructed spectrum *x*_1_ is consistent with the reconstructed spectrum (red line) in Figure 5(a). The reconstructed spectrum *x*_2_ is obtained by norm approximation optimization algorithm, where *ϖ* = 5.9% and *ϖ*_max_ = 18.4%. The norm approximation optimization algorithm is shown in Eq. (10), where *ε*_1_ = 130, *ε*_2_ = 10, and *ε*_3_ = 110 [29]. The reconstructed spectrum *x*_3_ obtained by least-squares optimization algorithm (the analytical solution is expressed as *x* = *P* ^*T*^ × (*P* × *P* ^*T*^)^−1^ × *D*) have relatively large errors (*ϖ* = 9.5% and *ϖ*_max_ = 32.1%) due to the absence of associated optimization items.

(10) $$\begin{array}{c} \min{\left\|P\times x-D\right\|}_{2}+\varepsilon_{1}{\left\|x\right\|}_{2}+\varepsilon_{2}{\left\|x\right\|}_{1}+\varepsilon_{3}{\left\|B\times x\right\|}_{2}\\ s.t.\;x\geq 0 \end{array}$$

### 4.4 Single wavelength characterization

Finally, to verify the system resolution, the laser signal with the center wavelength of 784.89 nm and FWHM of 0.17 nm is used as the system incident spectrum, and the reconstructed result (red line) by three MRRs is shown in Figure 6(b). The reconstructed spectrum center wavelength is 784.93 nm, and the center wavelength reconstruction error is 0.04 nm as shown in Figure 6(c). This error is mainly due to weight modulation and more accurate reconstructed spectrum will be obtained by increasing the number of MRRs appropriately.

Table 1 shows the comparison of the main schemes and technical parameters of on-chip spectrum detection systems reported in recent years. While achieving the high system resolution and operating bandwidth, the number of system physical and test channels directly affect loss and device footprint. The introduction of external tuning can effectively reduce the number of system physical channels, but in fact the test channels are not reduced, typically including split-channel spectrum detection with high Q-value MRR [30] and Fourier transform spectrum reconstruction based on MZI [20]. Therefore, we expect to use fewer system physical channels and test channels to implement spectrum detection systems with smaller device footprints. Based on the compressed sensing method, fewer system test channels can be used to characterize the unknown spectrum. Our proposed MRRAS achieves resolution better than 0.17 nm using only three MRR without external tuning, which means that spectrum can be reconstructed by acquiring only three sets of test data. However, limited by the linewidth of the employed laser, which is 0.17 nm, we cannot provide a reference spectrum with a narrower bandwidth to further characterize the system resolution. In the next step, we intend to extend the operating bandwidth by small radius micro-rings with high-quality spectrum and introducing subwavelength grating (SWG).

**Table 1:** Comparison of on-chip spectrum detection systems.

Ref	Spectrum	Resolution	Operating	Central	System physical/test	Integrated	External
	detection system		bandwidth	wavelength	channels	platform	tuning
2007 [31]	Split-channel: AWG	0.2 nm	20 nm	∼1545 nm	100/100	SOI	No
2019 [15]	Split-channel: AWG + MRR	0.1 nm	25.4 nm	∼1555 nm	9/254	SOI	Yes
2011 [27]	Split-channel: MRR	0.6 nm	50 nm	∼1580 nm	84/84	SOI	No
2022 [30]	Split-channel spectrometer: MRR	0.005 nm	10 nm	∼1550 nm	10/1740	SOI	Yes/50.4 mW
2013 [18]	Spatial heterodyne Fourier transform	0.04 nm	0.75 nm	∼1550 nm	32/—	SOI	No
2007 [23]	Stationary-wave integrated Fourier transform	4 nm	96 nm	∼1550 nm	1/—	SOI	No
2018 [24]	Stationary-wave integrated Fourier transform	4 nm	80 nm	∼1584 nm	1/—	Lithium niobate (LN)	Yes/<100 V
2019 [26]	Stationary-wave integrated Fourier transform with MRR-assisted	0.47 nm	90 nm	∼1571 nm	1/—	SOI	Yes/35 mW for MRR and 1.8 W for MZI
2021 [32]	Digital spectrometer by optimization algorithm with stratified waveguide filters	0.45 nm	180 nm	∼1550 nm	32/32	SOI	No
2020 [33]	Digital spectrometer by optimization algorithm with MRR array	0.08 nm	40 nm	∼1550 nm	8/400	Silicon nitride	Yes/25 V
2018 [29]	Digital Fourier transform spectrometer	0.2 nm	4.8 nm	∼1560 nm	1/64	SOI	Yes/99 mW
	Our work	<0.17 nm	>12 nm	∼809 nm	3/3	Silicon nitride	No

## 5 Conclusions

In conclusion, we have proposed an integrated on-chip spectrum detection scheme with a MRR array, which could construct the system transmission matrix and the system under-determined matrix equations. The excellent performance of the least-norm convex optimization algorithm for solving under-determined matrix equations is demonstrated in detail by theory and simulation. The experiment results show that the typical spectrum can be effectively reconstructed by using three MRRs with slightly varied radii. This experiment provides a scheme for the new on-chip spectrum detection system, which can reconstruct the unknown spectrum with different bandwidths on different waveguide material platforms. Compared with F-T spectrometer and split-channel spectrum detection system, MRRAS can reduce system complexity and avoid system power consumption caused by external modulation.

## 6 Fabrication and measurement

### 6.1 Silicon nitride waveguide device fabrication

First, a silicon nitride-on-SiO_2_ wafer is prepared with a 300 nm thick LPCVD-grown silicon nitride on a 3 μm thick SiO_2_ bottom cladding on a silicon substrate. Second, ∼400 nm thick e-beam resist (AR-P 6200.13) is deposited on top of the silicon nitride layer by spin-coating, we use the electron beam lithography (nB5) to define the pattern, followed by developing in *n*-amyl acetate for 2 min, and fixed it in isopropyl alcohol (IPA) for 5 min. Next, the silicon nitride layer is continuously etched for 4 min 15 s using the inductively coupled plasma tool (Plasma Pro 100 Cobra 300), and the gas composition is mainly SF_6_ and CHF_3_. Finally, the on-chip device pattern of the silicon nitride layer is obtained after cleaning the remaining resist and polymer with *N*-methyl-2-pyrrolidone (NMP) and piranha solution. Note that the silicon nitride waveguide width of 500 nm and the thickness of 300 nm is chosen based on the simulation results.

### 6.2 Measurement setup

In this work, the commercial spectrometer (YOKOGAWA-AQ6370D) is used to calibrate the system transmission matrix. In addition, the broadband spectrum from a super-continuum source (YSL SC-Pro) with an acousto-optic tunable filter (YSL AOTF) as an input spectrum was used to test the performance of the MRRAS. The narrowband spectrum from a laser source (Laser785-5HS) as an input spectrum was used to test the system resolution.
